# Clinical implications of a single *Staphylococcus lugdunensis*-positive blood culture: true bacteremia vs. contamination

**DOI:** 10.1128/jcm.00475-26

**Published:** 2026-07-27

**Authors:** A. Yogo, J. Krishnan, G. R. Lewin, L. S. Hojat, E. M. Ransom

**Affiliations:** 1 Department of Medicine, Case Western Reserve University School of Medicine 12304, Cleveland, Ohio, USA; 2 Division of Infectious Diseases and HIV Medicine, University Hospitals Cleveland Medical Center 114516, Cleveland, Ohio, USA; 3 Center for Global Health and Diseases, Case Western Reserve University School of Medicine 12304, Cleveland, Ohio, USA; 4 Department of Pathology, Case Western Reserve University School of Medicine 12304, Cleveland, Ohio, USA; 5 Case Western Reserve University-Cleveland VA Medical Center for Antimicrobial Resistance and Epidemiology 685904, Cleveland, Ohio, USA; 6 Division of Infectious Diseases, Emory University School of Medicine 12239, Atlanta, Georgia, USA; 7 Department of Pathology, University Hospitals Cleveland Medical Center 114516, Cleveland, Ohio, USA; Maine Medical Center Department of Medicine, Portland, Maine, USA

**Keywords:** *S. lugdunensis*, Staph lug, SOSA, blood culture, bacteremia, pseudobacteremia, contamination, contaminant, NHSN, IDSA

## Abstract

*Staphylococcus lugdunensis* is a common skin commensal. However, *S. lugdunensis* found in multiple blood culture sets is concerning for life-threatening bacteremia. When a single set is positive, the National Healthcare Safety Network classifies *S. lugdunensis* as a common commensal (contaminant), while multiple publications found bacteremia and suggested an Infectious Diseases (ID) consultation. Here, a retrospective review spanning 9 years at a large health system evaluated the clinical significance of a single-positive *S. lugdunensis* blood culture when multiple cultures were collected. *S. lugdunensis* was found in blood cultures from 137 patients, who were stratified into single (*n* = 91) and multiple (*n* = 46) set-positive groups. The single-positive group was more likely to be deemed contaminants by clinicians, less likely to have a diagnosis of endocarditis or catheter-related bloodstream infection, and less likely to have an intravascular device (*P* < 0.01). Treatment occurrence and duration were also statistically different (*P* < 0.01). Single-positive cultures were statistically more likely to be polymicrobial (*P* = 0.04). Additionally, each case was reclassified as significant bacteremia, indeterminate bacteremia, or contamination using the Souvenir criteria. In single-positive cultures, significant bacteremia occurred less often (12.1% vs. 43.5%, *P* < 0.01), while contamination was more common (81.3% vs. 32.6%, *P* < 0.01). Of note, nearly all significant bacteremia cases involved an intravascular device (96.8%). Taken together, these findings suggest single-positive *S. lugdunensis* blood cultures are often contaminants, but certain clinical features, including intravascular devices, strongly predict bacteremia.

**IMPORTANCE:**

A single-positive blood culture set with *Staphylococcus lugdunensis* can be difficult to distinguish between contamination and bacteremia. Conflicting clinical guidance has added to the confusion and emphasizes the need for additional studies. Our results suggest a single-positive *S. lugdunensis* blood culture set often reflects contamination and polymicrobial cultures, whereas multiple-positive sets strongly predict significant bacteremia. We also identified predictors of significant bacteremia, such as the presence of an intravascular device, emphasizing the need to incorporate additional metrics into clinical decision-making algorithms. Our findings underscore the role of clinical judgment when *S. lugdunensis* is recovered from a blood culture. Taken together, the proposed practice for the microbiology laboratory is to include a contamination comment on the report of a single-positive *S. lugdunensis* blood culture set and to recommend an infectious disease consultation for clinical adjudication.

## INTRODUCTION

*Staphylococcus lugdunensis* is a species of staphylococci other than *Staphylococcus aureus* (SOSA, previously termed coagulase-negative staphylococci), a group of bacteria typically regarded as a low-virulence member of the skin microbiota. However, *S. lugdunensis* is often considered of higher clinical significance than other SOSA. *S. lugdunensis* can demonstrate pathogenic behavior more akin to *S. aureus*, with the capacity to cause severe infections such as infective endocarditis, osteomyelitis, and deep tissue abscesses, often associated with significant morbidity and mortality. Therefore, when *S. lugdunensis* is recovered from blood cultures, there should be careful clinical evaluation, as it may represent a true and potentially serious infection (1, 2).

Interpretation of SOSA recovery from blood cultures can be challenging due to their dual role as common skin commensals (contaminants) and opportunistic pathogens. To better distinguish between true bacteremia and contamination for SOSA and other opportunistic pathogens, multiple advisory groups recommend collecting at least two blood culture sets, ideally from separate sites (3, 4). This recommendation is based on the logic that contamination is less likely when multiple sets are positive for a common skin commensal. Similarly, clinical microbiology laboratories often include a contaminant comment in blood culture reports to guide providers when common skin commensals are found in only one blood culture set.

However, guidance on interpreting single-positive *S. lugdunensis* blood cultures remains inconsistent. The U.S. Centers for Disease Control and Prevention National Healthcare Safety Network (NHSN) classifies *S. lugdunensis* as a common commensal (i.e., probable contaminant) when isolated from a single-positive blood culture set (5, 6). The European Manual of Clinical Microbiology similarly recommends withholding antimicrobial therapy in low-risk patients with a single-positive culture, citing the possibility of contamination (7). Additionally, a report has described favorable outcomes without treatment in such cases (8). However, it is not uncommon for health systems to require an Infectious Diseases (ID) consultation for a single-positive *S*. *lugdunensis* blood culture set. This practice is supported by a retrospective study, in which 13/29 patients (45%) with a single *S. lugdunensis*-positive blood culture had significant bacteremia based on the criteria of Souvenir et al., concluding *S. lugdunensis* in a single set of blood cultures often requires treatment (9). The Souvenir criteria classify cases as significant bacteremia, indeterminate bacteremia, or pseudobacteremia (contamination) based on major risk factors for potential infection caused by skin flora and clinical signs or laboratory findings (10). Importantly, the Souvenir criteria have previously demonstrated agreement with clinical impressions in classifying significant bloodstream infections from contamination (10, 11). Given the discrepancies in clinical practice, professional society guidelines, and published literature, this study conducted the largest retrospective chart and microbiological review of cases with *S. lugdunensis* identified in blood cultures to better characterize its clinical significance.

## MATERIALS AND METHODS

### Study design

The study was approved by the University Hospital Institutional Review Board, which waived the requirement for patient consent. This retrospective cohort study was conducted at a large integrated health system with a centralized microbiology laboratory. All hospitalized patients aged 18–89 years with a blood culture collected between January 1 2016, and December 13 2024, containing *S. lugdunensis* were included. All patients had at least two separate sets of blood cultures collected within 24 h. Clinical characteristics and outcomes were evaluated among those with a single set of positive blood cultures and those with two or more sets of positive blood cultures.

### Blood culture testing

Blood cultures were collected and tested following routine standard-of-care practices. Briefly, blood cultures were drawn into aerobic (bottle types: SA or FA plus) and anaerobic (bottle types: SN or FN plus) blood culture collection bottles (BacT/ALERT, bioMérieux, Marcy-l'Étoile, France) and incubated in the BacT/ALERT 3D or VIRTUO automated growth detection systems (bioMérieux, Marcy-l'Étoile, France). Incubation duration was 5 days from 2016 to 2020 and 4 days from 2020 to 2024. Gram stains were performed on positive blood cultures. Samples were plated to sheep’s blood, chocolate, MacConkey, and CDC anaerobic agar plates. Aerobic agars were incubated in the BD Kiestra Total Lab Automation System (BD Biosciences, Franklin Lakes, NJ, USA), and the BD GasPak EZ anaerobe pouch system with an indicator was used for anaerobic agars. The MALDI-TOF MS Biotyper (Bruker Scientific, Billerica, MA, USA) was used for microbial identification. Single-positive *S. lugdunensis* blood cultures were reported as “*Staphylococcus lugdunensis,*” underwent antimicrobial susceptibility testing, and did not have a test comment regarding contamination. Other single-positive SOSA isolates were reported as “Coagulase-negative staphylococcus,” did not undergo antimicrobial susceptibility testing, and had a contamination comment.

### Data collection

**Fig 1:**
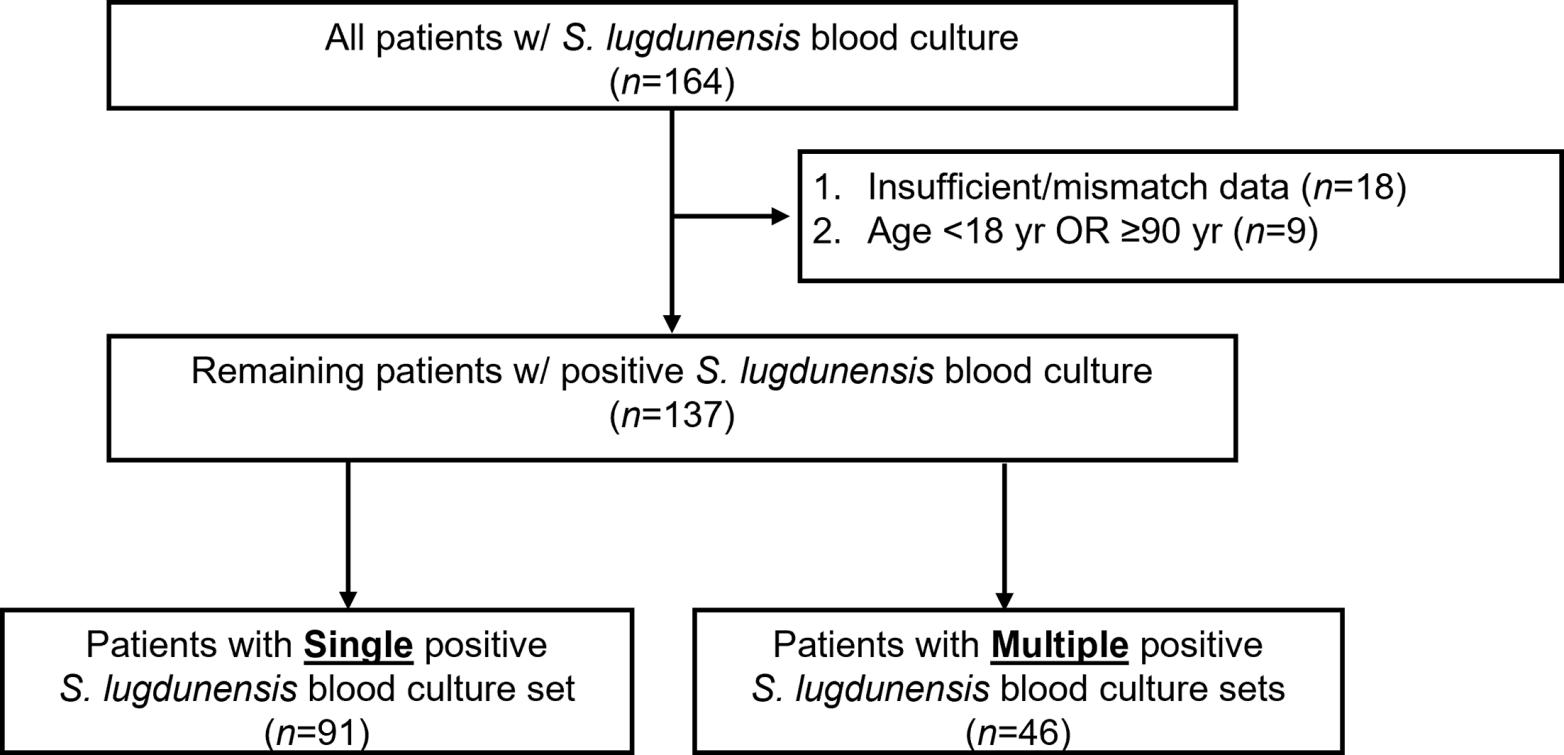
Patient selection workflow

A single blood culture set was defined as a single collection event consisting of one aerobic and one anaerobic bottle. Data from each patient, including demographics and comorbidities, were collected from an electronic data warehouse, a repository of archived electronic medical record data, and matched with blood culture data extracted from our microbiology laboratory information systems (SoftLab and Epic Beaker). Only patients with matching laboratory and patient records were included in the study (Fig. 1). If a patient had multiple encounters, only the initial encounter with the first blood culture was used for subsequent analyses. Additional data collected included age, sex, body mass index, Charlson Comorbidity Index, immunosuppressive status based on International Classification of Diseases (ICD) code (ICD Version 10 and ICD Codes: B20, B59, D45, D70.0-D70.9, D71, D80.0-D80.9, D81.0-D81.9, D82.0-D82.9, D83.0-D83.9, D84.0-D84.9, D89.0-D89.9, E41-E43, and Z94.0-Z94.9), intensive care unit admission, intravascular devices [e.g., implantable pacemaker and cardioverter-defibrillator, peripherally inserted central catheter (PICC), hemodialysis catheter (HD), and central catheters], presence of deep skin break (traumatic deep or puncture wound documented), infectious etiology per clinician documentation, ID consultation, follow-up blood cultures, transthoracic echocardiograms (TTE), transesophageal echocardiograms (TEE), duration of antimicrobial therapy, highest and lowest vital signs within 48 h of culture collection such as temperature, systolic blood pressure, heart rate, respiratory rate, and highest white blood cell count.

### Analysis

Descriptive statistics were used to analyze the characteristics of each patient group. Statistical significance was defined by a *P* value of <0.05 after multiple testing correction. Outcomes were compared using the Student’s *t* test for continuous variables and the Fisher’s exact test for categorical variables. All statistical analyses were performed in GraphPad Prism 10 or R version 4.5.0. Each group was retrospectively classified using the Souvenir criteria, which distinguishes significant bacteremia, indeterminate bacteremia, and pseudobacteremia (contamination) based on clinical and laboratory risk factors (Table 1) (10). Significant bacteremia is defined as having 1) one or more major risk factor(s) for potential infection caused by skin flora including long-term intravascular catheterization, immunosuppressed patients with central lines, peritoneal dialysis or hemodialysis patients, and patients with extensive postsurgical infections with SOSA PLUS 2) one or more clinical signs or laboratory findings: prolonged fever (≥38°C), hypotension (<90 mm Hg), leukocytosis (white blood cell count ≥ 12,000) or neutropenia with a left shift differential, or disseminated intravascular coagulopathy. Indeterminate bacteremia is defined as one major risk factor (above) with minimal clinical signs or symptoms. Pseudobacteremia is defined as not significant bacteremia and not indeterminate bacteremia.

**TABLE 1:** Souvenir criteria (10)

Significant bacteremia
One or more major risk factors for potential infection caused by skin flora: Long-term intravascular catheterization Immunosuppressed with central lines Peritoneal dialysis or hemodialysis Extensive postsurgical infection with SOSA
**Plus**
One or more clinical signs or laboratory findings: Prolonged temperature (≥38℃) Hypotension (<90 mm Hg) Leukocytosis or neutropenia with a left shift differential Disseminated intravascular coagulopathy

Indeterminate Bacteremia
One major risk factor (above) with minimal clinical signs or symptoms

Pseudobacteremia
Other than Significant and Indeterminate Bacteremia

## RESULTS

A total of 164 patients were identified with a blood culture containing *S. lugdunensis* during the study period. After the exclusion of ineligible patients based on age (*n* = 9) and data set limitations (*n* = 18), 137 patients were officially enrolled (Fig. 1). The median age was 65 y, and 49.6% of the population was male (Table 2). The median Charlson Comorbidity Index was 4, and 33 patients required admission to an intensive care unit (ICU). The median temperature was 37.0°C. Documented diagnoses were predominantly contamination (47.4%, *n* = 65), catheter-related bloodstream infection (CRBSI) (15.3%, *n* = 21), primary bacteremia (11.7%, *n* = 16), and endocarditis (11.7%, *n* = 16). ID consultations were performed in 71.5% (*n* = 98) of cases. TTE occurred in 61.3% of cases (*n* = 84), and TEE occurred in 17.5% of cases (*n* = 24). Importantly, treatment for *S. lugdunensis* was administered to 53.3% of patients (*n* = 73), and the median duration of therapy was 14 days. The overall mortality rates at 30 days and 90 days following blood culture collection were 17.0% and 18.2%, respectively.

**TABLE 2:** Characteristics of patients with *S. lugdunensis* in single or multiple blood cultures^*a*^

	Overall *n*= 137	Single *n*= 91	Multiple *n*= 46	Adj. *P* value
Age (years), median (IQR)	65 (53–77)	64 (52–77)	66 (54–74)	1.00
Male, *n* (%)	68 (49.6%)	46 (50.5%)	22 (47.8%)	1.00
Body mass index, median (IQR)	29.9 (24.7–38.7)	29.8 (24.4–39.3)	31.8 (25.3–38.4)	0.82
Charlson Comorbidity Index, median (IQR)	4 (2–7)	3 (2–7)	5 (2–8)	0.62
Immunocompromised host, *n* (%)	10 (7.3%)	7 (7.7%)	3 (6.5%)	1.00
Intensive care unit admission, *n* (%)	33 (24.1%)	21 (23.1%)	12 (26.1%)	1.00
Predisposing factor				
Intravascular device, *n* (%)	53 (38.7%)	21 (23.1%)	32 (69.6%)	<0.01
Deep skin break, *n* (%)	5 (3.6%)	4 (4.4%)	1 (2.2%)	1.00
Vital signs				
Fever (°C), median (IQR)	37.0 (36.5–37.6)	36.8 (36.4–37.4)	37.3 (36.6-38.0)	0.06
White blood cell count (10^9^/L), median (IQR)	11.4 (8.10-15.4)	11.7 (8.3-15.3)	11.1 (8.0-16.1)	0.88
Systolic blood pressure (mm Hg), median (IQR)	104 (92-121)	104 (93-120)	105 (90-123)	0.88
Heart rate (bpm), median (IQR)	99 (84-118)	99 (83-117)	101 (86-121)	0.67
Respiratory rate (breaths/min), median (IQR)	22 (18-27)	22 (18-27)	22 (20-28)	0.60
Endocarditis, *n* (%)	16 (11.7%)	3 (3.3%)	13 (28.3%)	<0.01
Catheter-related bloodstream infection, *n* (%)	21 (15.3%)	9 (9.9%)	12 (26.1%)	0.04
Skin and soft tissue infection, *n* (%)	10 (7.3%)	6 (6.6%)	4 (8.7%)	0.73
Other deep-seated foci, *n* (%)	9 (6.6%)	3 (3.3%)	6 (13.0%)	0.09
Primary bacteremia, *n* (%)	16 (11.7%)	12 (13.2%)	4 (8.7%)	0.67
Contamination, *n* (%)	65 (47.4%)	58 (63.7%)	7 (15.2%)	<0.01
Growth of multiple species, including *S. lugdunensis*, *n* (%)	52 (38.0%)	41 (45.0%)	11 (23.9%)	0.04
Follow-up blood culture, *n* (%)	102 (74.5%)	67 (73.6%)	35 (76.1%)	0.84
Echocardiography				
Transthoracic echocardiogram, *n* (%)	84 (61.3%)	46 (50.5%)	38 (82.6%)	<0.01
Transesophageal echocardiogram, *n* (%)	24 (17.5%)	7 (7.7%)	17 (37.0%)	<0.01
Infectious Diseases consultation, *n* (%)	98 (71.5%)	59 (64.8%)	39 (84.8%)	0.03
Antimicrobial treatment for *S. lugdunensis* infection, *n* (%)	73 (53.3%)	33 (39.5%)	40 (87.0%)	<0.01
Duration of therapy, median (IQR)	14 (0-28)	7 (0-14)	35 (14–42)	<0.01
Mortality				
30-day, *n* (%)	23/135 (17.0%)	13/90 (14.4%)	10/45 (22.2%)	0.38
90-day, *n* (%)	25/135 (18.2%)	14/90 (15.6%)	11/45 (24.4%)	0.32

^*a*^ Intravascular device: implantable pacemaker and cardioverter-defibrillator, peripherally inserted central catheter, hemodialysis catheter, and central catheter. Deep skin break: traumatic deep or puncture wound documented.

The 137 enrolled patients were classified as having a single-positive blood culture set (*n* = 91) or multiple-positive blood culture sets (*n* = 46) (Fig. 1). Patients with multiple-positive blood culture sets were more likely to be diagnosed with endocarditis (28.3% vs. 3.3%, *P* < 0.01), had increased occurrence of TTE (82.6% vs. 50.5%, *P* < 0.01) and TEE (37.0% vs. 7.7%, *P* < 0.01), and were more likely to have an intravascular device (69.6% vs. 23.1%, *P* < 0.01) (Table 2). CRBSI diagnoses were also increased in patients with multiple-positive *S. lugdunensis* blood culture sets (26.1% vs. 9.9%, *P* = 0.04), and other deep-seated foci neared statistical significance between groups (*P* = 0.09). ID consultations were increased in the patients with multiple-positive blood culture sets (84.8% vs. 64.8%, *P* = 0.03). Additionally, these patients had increased incidence of antimicrobial treatment (87.0% vs. 39.5%, *P* < 0.01) and median duration of treatment (35 days vs. 7 days, *P* < 0.01). Of note, higher fever was observed in this group of patients but was not found to be statistically significant (*P* = 0.06).

Cases of a single-positive blood culture set for *S. lugdunensis* were more likely to have a documented diagnosis of contamination (63.7% vs. 15.2%, *P* < 0.01), although using NHSN contamination criteria, 100% of the single-positive blood culture cases were deemed contaminated. Relatedly, these cultures were more likely to be polymicrobial (45.0% vs. 23.9%, *P* = 0.04). Of the 52 total polymicrobial cases across single- and multiple-positive blood cultures, non-commensal microorganisms were identified from the Souvenir pseudobacteremia group in 6 cases: 3 cases with *Klebsiella* species, one case with *Proteus mirabilis*, one case with *S. aureus*, and one case with *Candida* species. There was also one case with *Enterococcus faecalis* cocultured from the significant bacteremia group and one case with *Candida albicans* from the indeterminate bacteremia group. In the remaining 44 cases, the other identified species only included commensal microorganisms. However, 7 of these cases were classified as significant or indeterminate bacteremia by the Souvenir criteria. Chart review revealed the four significant bacteremia cases with polymicrobial commensal microorganisms included a CRBSI with leukocytosis, a clinical surgical site infection with shock in the setting of an intravascular catheter, an arteriovenous (AV) graft infection with multiple-positive *S. lugdunensis* blood culture sets, and an aortic valve replacement with multiple-positive *S. lugdunensis* blood culture sets. The three indeterminate cases with polymicrobial commensal microorganisms included a CRBSI with persistent *S. lugdunensis* bacteremia plus multiple-positive sets with other SOSA, a clinically suspected contamination with a single-positive *S. lugdunensis* blood culture, and a prosthetic valve endocarditis with two blood culture sets containing other SOSA.

According to the Souvenir criteria, significant bacteremia episodes occurred less often in patients from the single-positive set group (11 of 91 patients, 12.1%) than from the multiple-positive set group (20 of 46 patients, 43.5%; *P* < 0.01) (Table 3). On the other hand, pseudobacteremia occurred more often in patients from the single-positive set group (74 of 91 patients, 81.3%) than from the multiple-positive set group (15 of 46 patients, 32.6%; *P* < 0.01). Among clinically relevant predisposing factors, patients with significant or indeterminate bacteremia by the Souvenir criteria were more likely to have an intravascular device than patients with pseudobacteremia (*P* < 0.01) (Table 4). Chart review revealed that 30 of the 31 cases with true bacteremia had an intravascular device, including 20 with a PICC, HD, or central venous catheter (CV), and the rest with an AV graft or pacemaker/intracardiac defibrillator. In total, 53 patients in the study had intravascular devices including 34 with a PICC, HD, or CV that contributed to the Souvenir criteria schema. Also, using the Souvenir criteria, patients with significant or indeterminate bacteremia were less likely to have blood culture growth with multiple species, relative to patients with pseudobacteremia (*P* < 0.01). Importantly, clinical contamination correlated with pseudobacteremia (*P* < 0.01), and antimicrobial treatment for *S. lugdunensis* correlated with significant bacteremia (*P* < 0.01).

**TABLE 3:** Souvenir bacteremia classification in patients with *S. lugdunensis* in single or multiple blood cultures

	Overall *n*= 137	Single *n*= 91	Multiple *n*= 46	Adjusted *P* value
Significant bacteremia, *n* (%)	31 (22.6%)	11 (12.1%)	20 (43.5%)	<0.01
Indeterminate bacteremia, *n* (%)	17 (12.4%)	6 (6.6%)	11 (24.0%)	<0.01
Pseudobacteremia (contamination), *n* (%)	89 (65.0%)	74 (81.3%)	15 (32.6%)	<0.01

**TABLE 4:** Correlation of key metrics between bacteremia classification using Souvenir criteria^*a*^

	Significant bacteremia *n*= 31	Indeterminate bacteremia *n*= 17	Pseudobacteremia *n*= 89	Adjusted *P* value
Intravascular device, *n* (%)	30 (96.8%)	16 (94.1%)	7 (7.9%)	<0.01
Deep skin break, *n* (%)	0 (0.0%)	0 (0.0%)	5 (5.6%)	0.50
Growth with multiple species, *n* (%)	5 (16.1%)	4 (23.5%)	43 (48.3%)	<0.01
Clinical contamination, *n* (%)	1 (3.2%)	4 (23.5%)	60 (67.4%)	<0.01
Antimicrobial treatment for *S. lugdunensis* infection, *n* (%)	31 (100%)	13 (76.5%)	29 (32.6%)	<0.01

^*a*^ Intravascular device: implantable pacemaker and cardioverter-defibrillator, peripherally inserted central catheter, hemodialysis catheter, and central catheter. Deep skin break: traumatic deep or puncture wound documented. Growth with multiple species: polymicrobial positive blood cultures containing *S. lugdunensis *in combination with other organisms.

## DISCUSSION

SOSA isolates are often considered potential contaminants from a single-positive blood culture set and reported without antimicrobial susceptibility testing. The report may also include a contamination comment. These approaches are important for antimicrobial and diagnostic stewardship to prevent clinical teams from pursuing a potential red herring. However, there is mixed guidance on whether *S. lugdunensis* should be reported similar to other SOSA or more aggressively tested similar to *S. aureus*. Here, our 9-year retrospective review investigated the correlates and outcomes of blood cultures with *S. lugdunensis* using multiple approaches. Using the Souvenir criteria, we found a single-positive blood culture set with *S. lugdunensis* is likely to represent contamination compared to the multiple-positive set group (81.3% vs. 32.6%, *P* < 0.01), which may be underappreciated. Of patients categorized as having significant bacteremia by the Souvenir criteria, 96.8% had an intravascular device, while only 7.9% of those categorized as contamination had a device. In addition, blood cultures with multiple species were more associated with patients having pseudobacteremia (48.3%). Finally, 100% of patients categorized as having significant bacteremia underwent antimicrobial treatment for *S. lugdunensis* infection.

This is the largest study to date on *S. lugdunensis* blood culture interpretation, adding to the limited and conflicting literature. Fadel et al. demonstrated in a retrospective analysis that 45% of 29 patients with a single *S. lugdunensis*-positive blood culture had true bacteremia (9); however, in our study, it was only 12.1%. Sasaki et al. showed no significant differences in *S. lugdunensis* bacteremia between single-positive and multiple-positive cases, including for significant bacteremia (26.7% and 36.4%, respectively) and pseudobacteremia (65.0% and 54.5%, respectively) (8), in contrast to our findings. Similar to our findings, Non et al. demonstrated that isolating *S. lugdunensis* from two separate blood cultures is associated with a higher probability of significant bacteremia compared to isolating it from a single set (12). Thus, while our study substantially adds to our understanding of *S. lugdunensis* blood culture interpretation, these discrepancies emphasize the need for larger, multi-center studies.

Our data show that there were variable responses by clinicians to single-positive *S. lugdunensis* blood cultures. For instance, only 50.5% had a TTE, 73.6% of cases had follow-up blood cultures, and 64.8% had follow-up ID consultations. Importantly, 39.5% still received antimicrobial treatment for *S. lugdunensis* infection. The high level of variability in response to single-positive *S. lugdunensis* blood cultures may be driven by interpretation of laboratory results or differences in the clinical picture of patients, although it is also difficult to discern the impact of potentially biased literature/training that considers *S. lugdunensis* of higher significance than other SOSA. At our institution, single-positive *S. lugdunensis* blood culture reports do not include a contamination comment, in contrast to other SOSA organisms. Of note, this institutional guidance does not align with current policy from NHSN, which classifies *S. lugdunensis* as a common commensal and can be used by clinical laboratories to guide contamination comments. In addition, differences in responses are expected given that 12.1% of single-positive blood culture sets were deemed significant bacteremia by the Souvenir criteria. Our data support prior literature that suggests agreement between Souvenir criteria and clinical assessment in distinguishing true SOSA bacteremia from contamination, particularly noting Souvenir criteria’s high negative predictive value when clinical assessment is used as a gold standard (10). Accordingly, our study supports the clinical consensus that patients with blood cultures growing *S. lugdunensis* require more scrutiny compared to other SOSA species, due to its propensity to cause severe infections in humans akin to those of *S. aureus* (10–14). Given the importance of clinical decisions to distinguish true bacteremia from contamination (5, 6, 15), our findings support our proposed practice for the microbiology laboratory to use the contamination comment to align with NHSN but also recommend an ID consultation for clinical adjudication.

An ongoing challenge is that there is no definitive criterion—clinical or microbiological—for contamination, and clinicians ultimately must make the diagnosis based on clinical judgment. For example, Palavecino et al. have highlighted the variability in contamination reporting across clinical microbiology laboratories (16). Blood culture contamination rates and microorganism recovery can also vary by institution for a variety of reasons, such as collection techniques, blood fill volumes, and blood culture systems. Clinically, it is important to understand the factors that are more often associated with true bacteremia based on the Souvenir criteria. Among the clinical elements used to classify SOSA-positive blood cultures, long-term intravascular catheterization can be a predisposing factor for true bacteremia events (17, 18). Our data also show true bacteremia was more commonly associated with an intravascular device. This could represent a clinical predisposing factor that leads to the development of *S. lugdunensis* bacteremia, similar to other microorganisms that colonize the skin such as *S. aureus* (18, 19).

Another important consideration is growth of multiple species from the blood culture set. In our study, this metric was significantly more associated with pseudobacteremia (48.3%). However, 16.1% of the cases with significant bacteremia also grew multiple organisms. Knowing which other microorganisms were cocultured with *S. lugdunensis* adds value. For example, it is less convincing that *S. lugdunensis* is the causative agent of bacteremia when a second, more pathogenic microorganism is present (e.g., *Enterobacterales* and *Pseudomonas*). Also, a culture is likely to be considered contaminated if *S. lugdunensis* is found with other common commensal microorganisms (e.g., *Micrococcus* species, *Cutibacterium* species, and SOSA).

In conclusion, a single *S. lugdunensis*-positive blood culture set was more likely to represent contamination, whereas multiple-positive sets were more likely to be associated with true bacteremia. However, single-positive blood culture sets may still represent true bacteremia. These findings emphasize the need to balance antimicrobial stewardship with appropriate therapy and the utility of an ID consultation to investigate risk factors including intravascular devices and polymicrobial blood cultures.

## AFTER EPUB

*[This article was published on 27 July 2026 with an error in the text. The error was corrected in the current version, posted on 4 August 2026.]*
